# Tongue features of patients with granulomatous lobular mastitis

**DOI:** 10.1097/MD.0000000000031327

**Published:** 2022-11-18

**Authors:** Jiajing Chen, Jiyong Yang, Yuenong Qin, Chenping Sun, Jiatuo Xu, Xiqiu Zhou, Chunyu Wu, Yiyun Xu, Sheng Liu

**Affiliations:** a Department of Breast Surgery (Integrated Traditional and Western Medicine), Longhua Hospital affiliated to Shanghai University of Traditional Chinese Medicine, Shanghai, China; b Department of General Surgery, Longhua Hospital affiliated to Shanghai University of Traditional Chinese Medicine, Shanghai, China; c Shanghai University of Traditional Chinese Medicine, Shanghai, China.

**Keywords:** idiopathic granulomatous mastitis, inflammatory stress, TDA-1 tongue diagnostic and analysis system, traditional Chinese tongue diagnosis, white blood cells

## Abstract

Traditional Chinese tongue diagnosis plays an irreplaceable role in disease diagnosis. This study aimed to describe the tongue characteristics of patients with granulomatous lobular mastitis (GLM). Forty GLM patients and 40 non-GLM controls were evaluated using the Traditional Chinese Medicine subjective clinical interpretation and a TDA-1 Tongue Diagnostic and Analysis system. The associations between the image features of the tongue body and coating and the profiling of immune-inflammatory parameters were analyzed. GLM patients were prone to a reddish tongue bodies with thick, white, and greasy coatings. Thick and greasy tongue coating features are risk factors for GLM. GLM patients had higher levels of white blood cells (WBC), platelets, C-reactive protein, interleukin-2, and transforming growth factor-β (TGF-β) than non-GLM controls (*P* < .05). Also, tongue coating contrast and entropy values were significantly correlated with WBC or TGF-β levels in GLM patients (r < −0.310 and *P* < .05). We demonstrated that the hot evil and phlegm-dampness constitutions are the main characteristics of GLM. This might provide a reference for GLM diagnosis.

## 1. Introduction

Granulomatous lobular mastitis (GLM), also known as idiopathic granulomatous mastitis, is a rare and chronic inflammatory lesion of obscure etiology in the breast. GLM clinically mimics breast cancer and has been characterized by lobular necrosis, abscesses, skin ulcerations, painful multiple masses, and sinus formation.^[1]^ Most cases are of childbearing age with autoimmune diseases or a history of breastfeeding.^[2–4]^ The incidence of GLM is gradually increasing^[5]^ and the definitive histological diagnosis is the gold standard of GLM.^[2]^

Most GLMs are firstly diagnosed by preoperative investigations, including ultrasonography and imaging.^[6]^ However, clinical misdiagnosis often happens with this approach and the definitive diagnosis depends on the postoperative pathological method or preoperative invasive biopsy.^[6]^ Traditional Chinese tongue diagnosis (TCTD) is an important and main component of traditional Chinese medicine (TCM). TCTD plays an irreplaceable role in TCM diagnosis for thousands of years.^[7–9]^ TCM holds that GLM is due largely to lifestyle, diet, emotional disturbance, the presence of exogenous evils (including wind, cold, summer-heat, dampness, dryness, and fire), and the disorder of Chong-Ren meridian.^[10,11]^ The TCTD relies on the subjective opinion and experience-based inspection from experts. TCM practitioners probe the qi-blood and yin-yang disorders based on the features of the tongue bodies and coatings, the main criteria and vital parameters sensitively reflect the physiological and pathological conditions of the inter organs.^[7,9,12–15]^ Moreover, TCTD is an effective and noninvasive auxiliary method to be performed at any time, anywhere.^[12,15]^ However, traditional TCTD is subjective and can be easily influenced by factors like light and the external environment.

Fortunately, advances in automatic tongue diagnosis techniques have been made during the past decades with the development of computational-based digital processing techniques.^[7,15,16]^ The automatic tongue diagnosis system (ATDS) has been developed to provide a technical platform for the collection, storage, and quantification of a variety of features of tongue digital images.^[13,17]^ Accordingly, ATDS provides practitioners with objective information to assist and promote TCM diagnosis with a high agreement.^[7,15,17]^ Also, the tongue color and texture features or diagnostic models based on the ATDS have high accuracies in diagnosing specific diseases, including breast cancer, *Helicobacter pylori* infection, diabetes, and metabolic syndromes.^[9,15,17,18]^ However, the is poor information on the application and effect of ATDS in GLM diagnosis.

This study aimed to evaluate the efficiency of the ATDS image features in GLM diagnosis. The image features of the tongue bodies and coatings were obtained using the TDA-1 handheld tongue diagnostic instrument and TDA-1 Tongue Diagnostic and Analysis system (TDAS) designed by the Shanghai University of Traditional Chinese Medicine.^[17]^ This study will provide a theoretical basis and a reference for the application of intelligent tongue diagnosis technology in the clinical diagnosis of GLM.

## 2. Materials and methods

### 2.1. Participants

**Figure 1.**
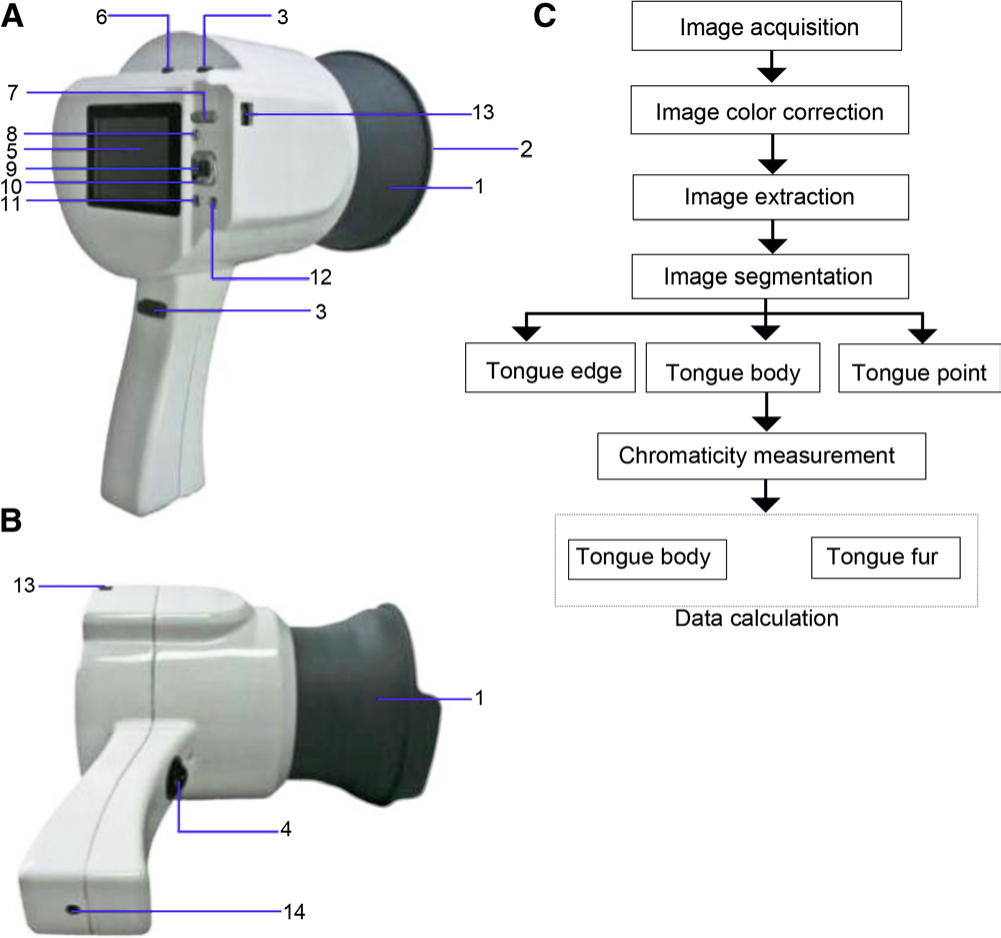
TDA-1 handheld tongue diagnostic instrument composition and traditional Chinese tongue diagnosis and Analysis system used in this study. (A and B) The TDA-1 handheld tongue diagnostic instrument composition. 1, collection ring; 2, UV mirror; 3, photo button; 4, camera module; 5, control pane; 6, camera switch; 7, focus shift button; 8, play back button; 9, OK button; 10, 4-way navigation button; 11, menu button; 12, scene button; 13, PC/AV port; 14, current input port. C, the traditional Chinese tongue diagnosis and Analysis system used in this study.

Eighty participants (GLM = 40 and non-GLM = 40) were collected from March 2017 to March 2018 from the Longhua Hospital Affiliated to Shanghai University of Traditional Chinese Medicine, Shanghai, China. The tongue images were collected from all individuals using the TDA-1 handheld tongue diagnostic instrument and TCTD system (version 2.0; Shanghai University of Traditional Chinese Medicine, Shanghai, China; Fig. 1).

GLM was diagnosed by at least 2 authors (doctors) using the postoperative pathological examination. Patients were included if they met the following inclusion criteria: GLM or idiopathic granulomatous mastitis patients who had not received any prior treatment for this study; 18 to 70 years old; willing to accept the clinical treatment programs of this study. Patients were excluded if they met the following exclusion criteria: previous use of hormones, immunosuppressive drugs, and/or had previous surgical treatment; with congenital cracked tongue, stripped moss, or other tongue surface abnormalities; pregnant women. Non-GLM controls were verified as healthy and disease-free through the hospital physical examination. This study is approved by the Ethics Committee of Longhua Hospital Affiliated to Shanghai University of Traditional Chinese Medicine, Shanghai, China. All patients were informed of the benefits and potential risks of the trial and signed informed consent.

### 2.2. TDA-1 digital tongue instrument and collection methods

The TDA-1 digital tongue imaging instrument (Fig. 1A and B) was employed to collect the tongue images of this study. The preoperative parameters were: manual mode, an aperture of F6. 3, shutter speed of 1/125 second, image ISO sensitivity of 200, no flash, and JPEG/BMP format. Data were collected with a built-in light source as previously reported by Zhang et al.^[17]^ In brief, subjects set down, looked ahead horizontally, and the chin was supported by the lower edge of the collection ring. The tongue body extended out approximately 1/2 to 2/3 and the tip of the tongue hung down naturally at a 60-degree angle to the horizon. The OK button was pressed to complete image acquisition. Image acquisition was repeated if necessary. Images were collected and analyzed using the TDAS (Shanghai University of Traditional Chinese Medicine)^[17,19]^ according to the process in Figure 1C.

### 2.3. The TDAS and chromatic indexes

The TDAS system included 3 basic functions (Display, Contrast, and History) and 3 main functions (extraction and segmentation of the tongue image, and measurement of chromaticity). The tongue images included 2 parts: the tongue body and tongue coating. We used the chromatic indexes of 3 color systems: RGB, CIEL*a*b*, and hospital information system (HIS), with the main indexes of RGB components (R for red, G for green, and B for blue], luminance (Y), chrominance blue (Cb), chrominance red (Cr), lightness/luminance (L*), red/green degree (a*), yellow/blue degree (b*), hue (H), brightness (I), and saturation (S).

The equations for converting RGB to HIS are listed as follows:


$$\begin{array}{l} L=\frac{1}{3}\left(R+G+B\right) \end{array}$$

$$\begin{array}{l} S=1-\frac{3}{\left(R+B+G\right)}\left[min\left(R,G,B\right)\right] \end{array}$$

$$\begin{array}{l} H=across\left\{\frac{\frac{1}{3}[(R-G)+(R-B)]}{{\left(R-G\right)}^{2}+(R-B){\left(G-B\right)}^{\frac{1}{2}}}\right\} \end{array}$$

For the texture characteristics of the tongue bodies and coatings, we used the following features: contrast (CON), angle inverse second moment (ASM), entropy (ENT), and mean (MEAN). The statistical gray difference was used to analyze the images. For a given image {f (i, j); i = 0, 1, 2, …, M-1; j = 0, 1, 2, …, N-1}; a smaller integer Δi, Δj was taken to obtain the gray difference CON. The ASM, ENT, and MEAN were all obtained using the following formulae:


$$\begin{array}{l} g(i,g)=f(i,j)-f(i+\Delta i,j+\Delta j) \end{array}$$

$$\begin{array}{l} MEAN=\frac{1}{m}\overset{}{\sum }ih_{g}\left(i\right) \end{array}$$

$$\begin{array}{l} CON=\sum_{i}i^{2}h_{g}(i) \end{array}$$

$$\begin{array}{l} ASM=\sum_{i}\left[h_{g}(i)\right] \end{array}$$

$$\begin{array}{l} ENT=-\overset{}{\sum }h_{g}\left(i\right)log_{2}\left(i\right) \end{array}$$

### 2.4. Tongue diagnosis in TCM and subjective clinical interpretation

Three TCM faculty members interpreted the images obtained from all GLM patients and non-GLM controls. The results of artificial interpretation are based on the diagnostic criteria of TCM. The tongue description was categorized into different categories according to the 9 primary features listed in Table 1.^[20]^ If there is a dispute, a fourth accountable TCM expert criticized and determined the diagnosis.

**Table 1:** Tongue features used in this study.

Features	Categories
Tongue shape	Small and thin, moderate, fat
Tongue color	Light red, red, dark red
Tongue coating color	None, white, yellow
Coating thickness	Thin, thick
Coating texture	Greasy, normal
Prick tongue	Yes, no
Tooth-marked tongue	Yes, no
Sublingual vessel	Yes, no
Ecchymosis	Yes, no

### 2.5. Hematology examination

The blood tests were performed on GLM patients and non-GLM controls at the Laboratory of the Longhua Hospital Affiliated to Shanghai University of Traditional Chinese Medicine. An automatic biochemical instrument was used to detect the levels of white blood cells (WBC), C-reactive protein (CRP), interleukin-2 (IL-2), platelets (PLT), transforming growth factor-β (TGF-β), and IL-6 in the whole blood sample.

### 2.6. Statistical analysis

All statistical analyses were conducted using the SPSS software (version 21.0, Chicago, IL). If the data followed an abnormal distribution, data were expressed as the median and range, the non-parametric Mann–Whitney *U* test was used to compare the data between 2 groups, or otherwise, the 2-tailed *t* test was applied. Counting variables were expressed as number and percent and the differences between groups were analyzed using the chi-square test or the Wilcoxon rank sum test. Tongue features associated with the incidence of GLM were identified using the univariate logistic regression analysis. The 95% confidence interval (CI) and odds ratio (OR) were calculated for the logistic regression analysis. Spearman correlation analysis was conducted to identify significant relationships between parameters. We evaluated the accuracy of a related factor in diagnosing GLM using the receiver operating characteristic curve (ROC). For all statistical tests, the significance level of alpha 0.05 (*P* < .05) was used.

## 3. Results

### 3.1. Demographics

The average ages of GLM patients and non-GLM controls were 34.10 ± 11.03 years old and 33.13 ± 8.47 years old, respectively (*P* = .660; Table 2), The average disease course of GLM patients was 7.80 ± 2.68 months. Compared with non-GLM controls, GLM patients had higher levels of WBC (8.85 vs 7.42 10^9^/L, *P* = .034), PLT (291.50 vs 205.00 10^9^/L, *P* = 3.689e-07), CRP (6.73 vs 2.69 mg/L, *P* = .104), IL-2 (112.80 vs 60.64 pg/mL, *P* = .019), IL-6 (3.15 vs 2.98 pg/mL, *P* = .500), and TGF-β (430.00 vs 186.08 pg/mL, *P* = 3.670e-08; Table 2). These results indicated that GLM patients showed increased inflammation.

**Table 2:** Demographics of patients with granulomatous lobular mastitis (GLM) and non-GLM healthy individuals.

Variables	GLM (n = 40)	Non-GLM (n = 40)	*P* value
Age (yr)	34.10 ± 11.03	33.13 ± 8.47	.660^#^
WBC (10^9^/L)	8.85 (4.70–21.14)	7.42 (6.19–8.67)	.034^ϕ^
PLT (10^9^/L)	291.50 (121.00–552.00)	205.00 (108.00–292.00)	3.689e-07^ϕ^
CRP (mg/L)	6.73 (0.50–85.63)	2.69 (1.73–3.88)	.104^ϕ^
IL-2 (pg/mL)	112.80 (8.50–262.70)	60.64 (46.07–76.05)	.019^ϕ^
IL-6 (pg/mL)	3.15 (2.00–26.40)	2.98 (1.61–4.66)	.500^ϕ^
TGF-β (pg/mL)	430.00 (51.70–1169.90)	186.08 (148.10–222.48)	3.670e-08^ϕ^
Tongue shape			2.000e-06^‡^
Small and thin (n, %)	0	9 (22.50%)	
Fat (n, %)	13 (32.50%)	6 (15.00%)	
Moderate (n, %)	27 (67.50%)	25 (62.50%)	
Tongue coating color			1.000e-06^‡^
Yellow (n, %)	2 (5.00%)	5 (12.50%)	
White (n, %)	32 (80.00%)	7 (17.50%)	
None (n, %)	6 (15.00%)	28 (70.00%)	
Greasy coating (n, %)	38 (95.00%)	6 (15.00%)	6.410e-13^†^
Thick tongue coating (n, %)	34 (85.00%)	5 (12.50%)	8.779e-11^†^
Tongue body color			2.349e-17^‡^
Light red (n, %)	40 (100.00%)	29 (72.50%)	
Red (n, %)	0	11 (27.50%)	
Dark red (n, %)	0	0	
Tooth-marked tongue (n, %)	15 (37.50%)	6 (15.00%)	.022^†^
Prick tongue (yes/no)	0	0	/
Sublingual vessel (yes/no)	15 (37.50%)	0	1.700e-05^†^
Ecchymosis (yes/no)	0	0	/

Special characters #, †, ‡, and ϕ following *P* value indicate differences analyzed by the 2-tailed *t* test, the chi-square test (progressive significance), Wilcoxon rank sum test, and Mann–Whitney *U* test, respectively.

CRP = C-reactive protein, GLM = granulomatous lobular mastitis, IL-2 = interleukin-2, IL-6 = interleukin-6, PLT = platelet, TGF-β = transforming growth factor-β, WBC = white blood cells.

### 3.2. Subjective clinical interpretation

**Figure 2.**
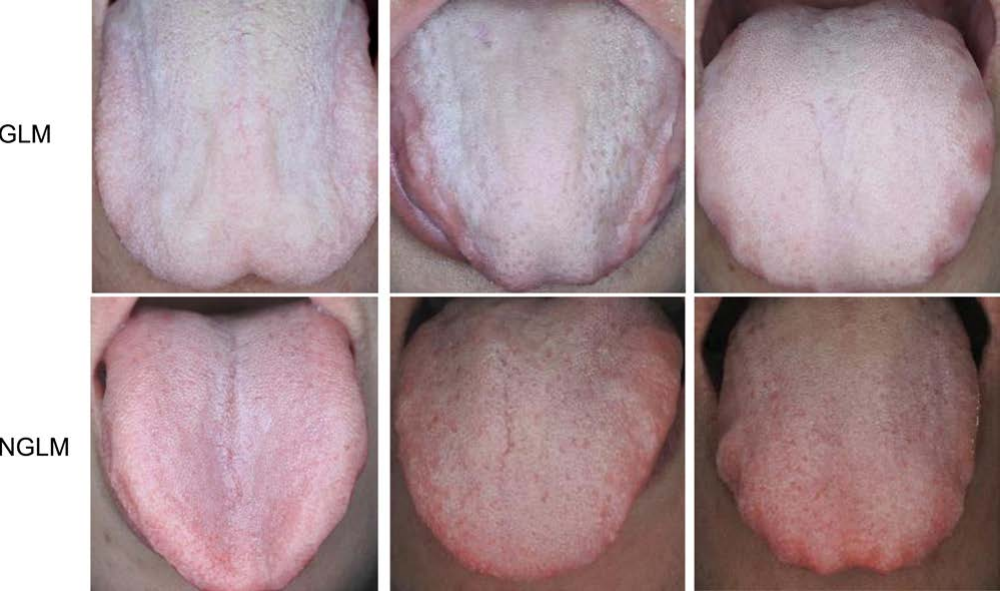
Representative tongue images. Representative tongue images were collected from 3 randomly selected granulomatous lobular mastitis (GLM) patients and 3 non-GLM (NGLM) healthy controls.

The clinical interpretation based on the tongue images showed that most GLM patients had white/yellow (85.00% vs 30.00%, progressive significance *P* = 1.000e-06), greasy (95.00% vs 15.00%, progressive significance *P* = 6.410e-13), and thick (85.00% vs 12.50%, progressive significance *P* = 8.779e-11) tongue coatings compared with non-GLM controls (Fig. 2). Also, GLM patients had a higher percent of tooth-marked (37.50% vs 15.00%, progressive significance *P* = .022), light red (100.00% vs 72.50%, *P* = 2.349e-17), and fat (32.50% vs 15.00%, *P* = 2.00e-06) tongue bodies compared with non-GLM controls (Table 2). These results indicated that white, thick, and greasy coatings and light red tongue bodies might be the tongue characteristics of GLM patients.

### 3.3. Tongue features associated with GLM incidence

**Figure 3.**
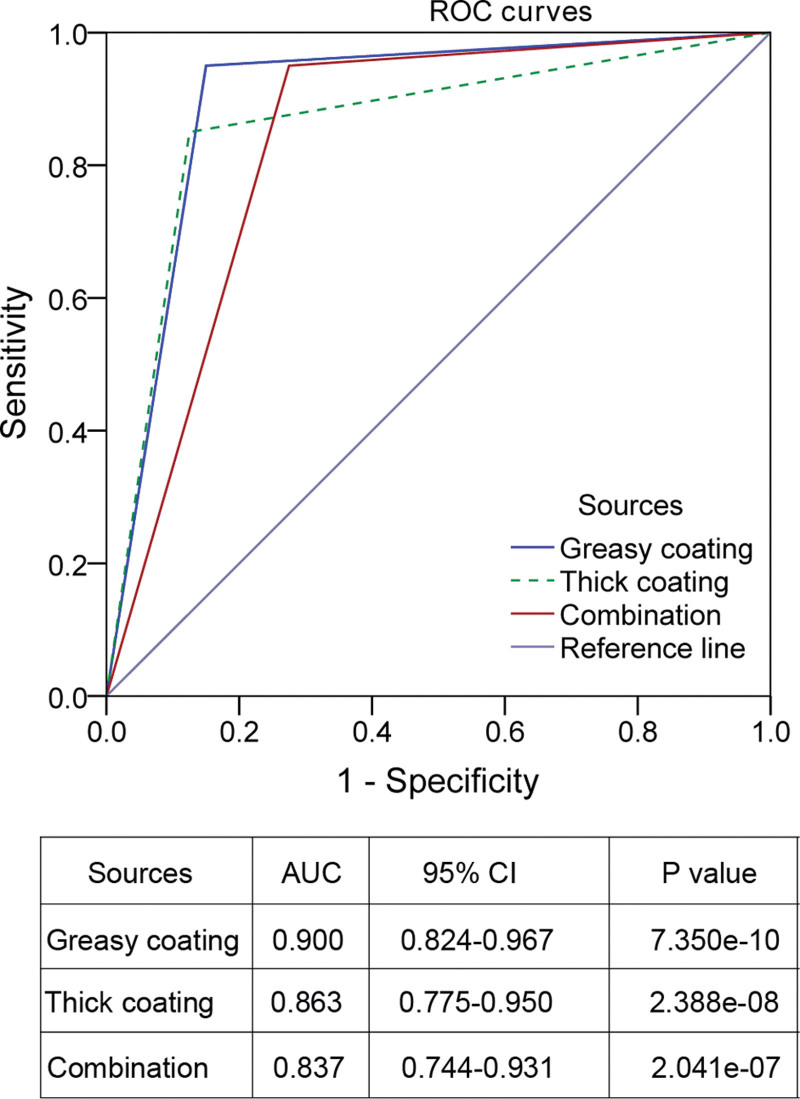
The receiver operating characteristic (ROC) curve analysis of coating features in diagnosing granulomatous lobular mastitis. AUC = area under the ROC curve, CI = confident interval.

We used the logistics regression analysis to identify the tongue features associated with GLM incidence. Univariate logistics analysis showed that white (OR = 18.857, 95% CI 6.123–58.079, *P* = 3.100e-07), greasy (OR = 107.667, 95% CI 20.352–569.573, *P* = 3.688e-08), and thick tongue coatings (OR = 39.997, 95% CI 11.060–142.270, *P* = 1.616e-08) and tooth-marked tongue bodies (OR = 3.778, 95% CI 1.291–11.057, *P* = .015) were related to higher incidence of GLM (Table 3). Further multivariate logistics regression analysis indicated that subjects with greasy and thick tongue coatings were at high risk of GLM (greasy coating: OR = 35.900, 95% CI 4.800–268.508, *P* = 4.875e-04; thick coating: OR = 17.644, 95% CI 2.772–112.288, *P* = 2.372e-04; Table 3). The ROC curve analysis showed that greasy coating had a higher accuracy in predicting GLM in this study cohort (area under the ROC curve, AUC = 0.900, 95% CI 0.824–0.967, *P* = 7.350e-10) compared with thick coating (AUC = 0.863, 95% CI 0.775–0.950, *P* = 2.388e-08) and the combination of them both (AUC = 0.837, 95% CI 0.744–0.931, *P* = 2.041e-07; Fig. 3).

**Table 3:** Tongue features associated with granulomatous lobular mastitis in this study by logistics analysis.

Tongue features		Univariate		Multivariate		
	OR	95% CI	*P*	OR	95% CI	*P*
Yellow coating	0.179	0.020–1.612	.125			
White coating	18.857	6.123–58.079	3.100e-07	4.134	0.665–25.704	.128
Greasy coating	107.667	20.352–569.573	3.688e-08	35.900	4.800–268.508	4.875e-04
Thick coating	39.997	11.060–142.270	1.616e-08	17.644	2.772–112.288	2.372e-04
Fat tongue	0.367	0.123–1.092	.071			
Tooth-marked tongue	3.778	1.291–11.057	.015	3.854	0.256–58.053	.330

CI = confident interval, OR = odds ratio.

### 3.4. Association of chromatic indexes in tongue images with GLM

The chromatic indexes of the RGB, CIE L*a*b*, and HIS systems in the tongue images of all participants were extracted and analyzed based on the TDAS system. We observed that the tongue bodies and coatings of GLM patients both had higher levels of I, G, B, L*, Y, Cb, and ASM compared with those of the control subjects (Table 4). Besides, the levels of S, CON, a*, b*, Cr, ENT, and MEAN of the GLM patients were lower than those of the non-GLM controls (Table 4). We also observed that the coating R level in GLM patients was significantly higher than that of the non-GLM controls (196.50 vs 168.50, *P* = 9.125e-05), but was lower in the tongue body of GLM patients (158.00 vs 163.50, *P* = .211). Logistics regression analysis showed that all chromatic indexes were associated with GLM (*P* < .05; See Supplemental Digital Content Table S1, http://links.lww.com/MD/H702).

**Table 4:** Comparison of chromatic indexes of the RGB, CIE Lab, and HL(I)S systems in the tongue body and tongue coating between granulomatous lobular mastitis (GLM) patients and non-GLM controls.

	Tongue body			Tongue coating		
Indexes	GLM	Non-GLM	*P* value	GLM	Non-GLM	*P* value
H	348.39 (0–358.91)	352.59 (0–359.14)	.044	323.46 (0–350.63)	332.65 (0–356.90)	.224
I	165.50 (99.00–216.00)	130.50 (102.00–170.00)	2.241e-05	179.00 (74.00–231.00)	147.00 (107.00–177.00)	3.052e-06
S	0.12 (0.08–0.21)	0.14 (0.0–0.21)	1.116e-04	0.07 (0.04–0.18)	0.09 (0.05–0.17)	.015
R	158.00 (80.00–208.00)	163.50 (135.00–201.00)	.211	196.50 (100.00–240.00)	168.50 (135.00–194.00)	9.125e-05
G	197.50 (132.00–245.00)	115.00 (85.00–152.00)	5.852e-14	166.00 (61.00–220.00)	138.50 (93.00–166.00)	3.038e-06
B	148.00 (78.00–198.00)	117.50 (85.00–159.00)	1.516e-04	176.50 (61.00–235.00)	135.00 (89.00–175.00)	1.417e-06
L*	119.33 (96.78–132.24)	109.20 (98.53–120.58)	3.976e-05	123.42 (86.83–136.12)	115.23 (101.29–123.18)	3.685e-06
a*	14.87 (11.05–24.42)	17.88 (11.47–22.68)	7.056e-04	8.42 (4.46–18.34)	10.43 (5.86–16.80)	2.045e-04
b*	1.11 (0.01–7.75)	4.50 (0.54–10.69)	6.211e-07	−2.76 (−5.62–6.67)	1.74 (−4.01–8.55)	1.176e-06
Y	171.84 (132.19–198.58)	126.89 (103.04–159.61)	2.569e-11	167.85 (78.40–211.45)	142.03 (107.80–166.4)	4.432e-06
Cr	148.81 (145.14–155.47)	150.99 (145.35–157.27)	9.642e-04	138.52 (132.85–145.13)	142.13 (135.11–150.61)	2.400e-05
Cb	125.20 (122.47–127.62)	121.39 (116.46–125.28)	2.411e-10	129.94 (121.80–140.51)	124.57 (118.41–131.18)	3.399e-07
CON	68.91 (11.07–121.53)	81.89 (41.79–162.86)	.018	80.06 (26.40–161.65)	124.90 (43.75–262.26)	4.319e-05
ASM	0.08 (0.06–0.23)	0.07 (0.05–0.11)	4.255e-03	0.07 (0.05–0.14)	0.06 (0.04–0.10)	1.625e-05
ENT	1.19 (0.75–1.33)	1.24 (1.09–1.40)	6.657e-03	1.23 (0.99–1.40)	1.34 (1.10–1.51)	2.844e-05
MEAN	0.02 (0.01–0.03)	0.03 (0.02–0.04)	8.861e-03	0.03 (0.02–0.04)	0.03 (0.02–0.05)	1.635e-05

All the differences were analyzed using the non-parametric Mann–Whitney *U* test.

a* = red/green degree, ASM = angle inverse second moment, b* = yellow/blue degree, Cb = chrominance blue, CON = contrast, Cr = chrominance red, ENT = entropysee, GLM = granulomatous lobular mastitis, H = hue, I = brightness, L* = lightness/luminance, MEAN = mean, S = saturation, Y = luminance.

### 3.5. Correlation of chromatic indexes in tongue images with inflammatory factors in GLM patients

Since the etiology of GLM is closely related to the dysfunction of the immune system and there were significant differences in inflammatory factors and chromatic indexes in GLM patients, we analyzed the correlation between inflammatory factors (WBC, CRP, IL-2, PLT, TGF-β, and IL-6) and the TDAS chromatic indexes in both the tongue bodies and coatings. Spearmen correlation analysis revealed that the RGB component Y, the HIS component H, the texture characteristics CON and ENT were associated with the levels of WBC and TGF-β in the blood samples of GLM patients (Table 5). TGF-β was negatively correlated with Y (correlation coefficient, r = −0.318, *P* = .045) and H (r = −0.395, *P* = .014). WBC count in GLM patients was correlated with Y (r = −0.367, *P* = .020), CON (r = −0.319, *P* = .045), and ENT (r = −0.329, *P* = .038).

**Table 5:** Correlations (Spearmen) between chromatic indexes in tongue images and inflammatory factors in the blood samples of granulomatous lobular mastitis patients.

Chromatic indexes	WBC		TGF-β	
	*r*	*P* value	*r*	*P* value
Tongue body				
Y	−0.367	.020	−0.318	.045
Tongue coating				
CON	−0.319	.045	/	/
ENT	−0.329	.038	/	/
H	/	/	−0.395	.014

CON = contrast, ENT = entropysee, H = hue, OR = odds ratio, TGF-β = transforming growth factor-β, WBC = white blood cells, Y = luminance.

## 4. Discussion

The effectiveness of ATDS in disease diagnosis has been supported by increasing evidence.^[7,15,16]^ The features of ATDS digital images provide practitioners with objective information to assist TCM diagnosis. Our present study showed that the GLM patients had significant changes in the digital image features of the RGB, CIE L*a*b*, and HIS systems as compared to non-GLM controls. Also, the Y, H, CON, and ENT indexes were significantly correlated with blood WBC count and TGF-β levels in GLM patients.

Although the pathogenesis of GLM is unknown, 3 hypotheses have been proposed regarding its etiology: autoimmune dysfunction, infection, and systemic endocrine disorders.^[21]^ Granulomatous inflammation can be caused by fungal, parasitic, and bacterial infections.^[22–24]^ For instance, *Corynebacterium* is the most common bacterium to cause abscessed breast granuloma.^[25–27]^ Also, there is supporting evidence for the hypothesis that GLM is a chronic inflammatory disorder that is associated with the activation, infiltration, and proliferation of T cells, neutrophils, and macrophages, among others.^[24,28]^ The above causes result in elevated inflammatory cytokines, leaving the body in a state of inflammatory stress, and ultimately causing the pathogenesis of inflammatory diseases.^[3,24]^ This study showed that patients with GLMs had higher levels of WBC, PLT, CRP, IL-2, and TGF-β compared with non-GLM controls, showing the increased inflammatory stress in GLM patients compared with non-GLM controls. Although the levels of these parameters were within a normal range in most GLM patients, the higher levels of them in GLM patients indicated that they were in a state of imbalanced immune function and inflammatory stress.

Immune-inflammatory parameters CRP, WBC, PLT, IL-2, IL-6, and TGF-β play important roles in inflammatory responses and immunity.^[29–32]^ For instance, IL-2 functions as a thymic regulatory T (Treg) cell growth factor and enhanced IL-2 signaling promotes Treg cell development.^[33,34]^ TGF-β is a multifunctional protein that affects the growth, differentiation, apoptosis, and immune regulation of many cells.^[31,35]^ TGF-β also promotes the production of human fibroblast IL-6 and inhibits epithelial cell growth.^[36]^ WBCs, including neutrophils, monocytes, lymphocytes, and PLT play remarkable roles in innate immune function by regulating inflammation and immune responses.^[37]^ Moreover, WBC and CRP could imply the progression of coronavirus disease 2019 (COVID-19) characterized by the overwhelming immune inflammatory response and cytokine storm.^[30,38]^ However, our present study demonstrated that most GLM patients had normal CRP, WBC, PLT, IL-2, IL-6, and TGF-β levels, which might prone to the negligence of inflammation in GLM.

TCTD is an important component of TCM and has played a remarkable role in TCM diagnosis for thousands of years.^[7–9]^ Therefore, we hypothesized that TCTD can be used as a supplementary and confirmatory system for GLM diagnosis. Using TCTD diagnosis, we demonstrated that most GLM patients had thick, white, and greasy tongue coatings and reddish tongue bodies. Moreover, we demonstrated that thick and greasy tongue coating were risk factors of GLM. TCM diagnosis holds that a reddish tongue body is related to hot evil qi (Re Xie) and the white and greasy tongue coating is associated with a phlegm-dampness constitution.^[39,40]^ Also, the higher the CON, ENT, and MEAN values, or the ASM value, the better the appearance of tongue coating is.^[41]^ We demonstrated that the tongue coating features CON, ENT, and MEAN in GLM patients were significantly lower than those in healthy controls, proving that GLM patients were prone to greasy, white, and thick tongue coatings. These results might indicate that phlegm-dampness is the main constitution type in GLM patients.

TCM practitioners have different understandings and experience-based opinions regarding the appropriate TCM treatment for GLM. Many TCM doctors only note white, thick, greasy tongue coatings and treat phlegm-dampness syndrome with herbs pungent in taste and warm in nature.^[42,43]^ However, the above treatments often ignore the hot evil qi behind the tongue coating and therefore the effects are not very obvious. We observed that most GLM patients had reddish tongue bodies (hot evil qi), showing that treatment with herbs cold in nature might be effective for GLM. This finding might provide a reference for making treatment strategies for GLM regarding the status of inflammatory stress and the characteristics of qi and blood in GLM patients.

Tongue diagnosis is an indispensable part of TCM diagnosis and can reflect the severity and development of diseases.^[44,45]^ The value of the TCTD diagnostic method is evident in diagnosing a variety of diseases, including breast cancer, gastrointestinal disorders, COVID-19, and diabetes.^[17,44–47]^ For instance, the proportion of greasy coatings was higher in patients with critical COVID-19 pneumonia compared patients with mild, moderate, and severe COVID-19 pneumonia.^[44]^ The amount and thickness of tongue coatings in the spleen–stomach area are related to the severity of gastroesophageal reflux disease.^[45]^ Using the TDAS system, we demonstrated that there were significant differences in the RGB, CIEL*a*b*, and HIS parameters in the tongue bodies and coatings of GLM patients compared with healthy controls. We observed that GLM patients had higher tongue coating G, B, I, Y, and Cb values and lower tongue coating R, S, a*, b*, CON, ENT, and MEAN. Also, the tongue coating CON, ENT, and H levels were negatively correlated with WBC and TGF-β levels in GLM patients. The tongue coating features might reflect the increasing inflammatory stress in GLM patients. Given these findings, our study might provide a reference for diagnosing GLM and assessing the status of inflammation stress in GLM patients based on the TDAS features.

## 5. Conclusion

In conclusion, our present study demonstrated that most GLM patients had reddish tongue bodies with thick, white, and greasy tongue coatings. These results showed that patients with GLMs were prone to hot evil qi and phlegm-dampness constitutions. We also demonstrated that almost all RGB, CIE L*a*b*, and HIS digital features in GLM patients were different to those in healthy controls. The tongue coating H, CON, and ENT were negatively correlated with blood WBC count and TGF-β levels in GLM patients. These results might provide a reference for GLM diagnosis and for making treatment strategies for GLM regarding the status of inflammatory stress and the characteristics of qi and blood in GLM patients combing the TDAS features.

## Author contributions

**Conceptualization:** Jiajing Chen, Sheng Liu.

**Data curation:** Jiajing Chen, Jiyong Yang, Yuenong Qin, Chenping Sun, Xiqiu Zhou, Yiyun Xu, Sheng Liu.

**Formal analysis:** Jiajing Chen, Jiyong Yang, Yuenong Qin, Chenping Sun, Xiqiu Zhou, Chunyu Wu, Yiyun Xu.

**Funding acquisition:** Sheng Liu, Xiqiu Zhou, Yuenong Qin.

**Investigation:** Jiajing Chen, Jiyong Yang, Yuenong Qin, Jiatuo Xu, Xiqiu Zhou, Sheng Liu.

**Methodology:** Jiyong Yang, Xiqiu Zhou, Chunyu Wu.

**Project administration:** Chunyu Wu, Sheng Liu.

**Resources:** Jiyong Yang, Yuenong Qin, Jiatuo Xu, Xiqiu Zhou, Chunyu Wu, Yiyun Xu.

**Software:** Yuenong Qin, Jiatuo Xu.

**Writing – original draft:** Jiajing Chen, Jiyong Yang.

**Writing – review & editing:** Yuenong Qin, Chenping Sun, Jiatuo Xu, Xiqiu Zhou, Chunyu Wu, Yiyun Xu, Sheng Liu.

## Supplementary Material
