# Supplementary material for: Tongue features of patients with granulomatous lobular mastitis
Source: Medicine (Baltimore). 2022 Nov 18;101(46):e31327. doi: 10.1097/MD.0000000000031327 (PMC9678557; doi:10.1097/MD.0000000000031327)
Supplement: Supplementary file 1 [file medi-101-e31327-s001.pdf]

Table S1. Association of chromatic indexes with granulomatous lobular mastitis analyzed by univariate logistics analysis.

Tongue body				Tongue coating		
Indexes	OR	95% CI	P value	OR	95% CI	P value
H	1.004	1.000-1.008	0.037	1.003	1.000-1.006	0.049
I	1.041	1.021-1.061	6.200e-05	1.040	1.020-1.061	1.043e-04
S	1.887e-14	2.453e-22-1.000e-06	0.001	6.288e-10	1.617e-18-0.245	0.036
R	0.984	0.968-1.000	0.050	1.036	1.015-1.058	0.001
G	1.169	1.062-1.286	0.001	1.041	1.020-1.063	1.000e-04
B	1.034	1.016-1.053	2.277e-04	1.037	1.019-1.056	4.062e-05
L*	1.135	1.056-1.209	8.300e-05	1.132	1.058-1.212	3.089e-04
a*	0.752	0.626-0.902	0.002	0.700	0.556-0.881	0.002
b*	0.615	0.484-0.781	7.000e-05	0.701	0.592-0.830	3.800e-05
Y	1.122	1.067-1.179	6.000e-06	1.048	1.023-1.073	1.310e-04
Cr	0.745	0.620-0.895	0.002	0.699	0.584-0.838	1.018e-04
Cb	3.092	1.853-5.161	1.600e-05	1.437	1.223-1.687	1.000e-05
CON	0.980	0.963-0.996	0.015	0.975	0.962-0.988	2.424e-04
ASM	5.800e+17	1.000e+5-3.367e+30	0.006	2.040e+31	3.424e+14-1.210e+48	2.536e-04
ENT	0.001	2.000e-06-0.104	0.005	1.300e-05	3.447e-08-0.005	1.745e-04
MEAN	6.080e-60	2.147e-102-1.718e-19	0.006	9.945e-95	1.028e-117-9.577e-40	8.800e-05

OR, odds ratio. CI, confident interval.
